# Prenatal diagnosis of pectus excavatum

**DOI:** 10.4274/tjod.54514

**Published:** 2016-09-15

**Authors:** Cihan Çetin, Selim Büyükkurt, Mete Sucu, Mehmet Özsürmeli, Cansun Demir

**Affiliations:** 1 Çukurova University Faculty of Medicine, Department of Obstetrics and Gynecology, Adana, Turkey

**Keywords:** Funnel chest, Prenatal diagnosis, ultrasonography, prenatal

## Abstract

Pectus excavatum (PE) is the depression of the lower part of manubrium sterni and xiphoid process. The main problem of PE depends on the cardiopulmonary morbidity caused by the narrowing of the thoracic space. To date, prenatal diagnosis of this deformity has been reported only once and was associated with Down syndrome. We present another case which we diagnosed as PE during a second-trimester fetal anatomic scan. The pectus severity index is used for these patients in postnatal life; however, prenatal adaption of this index is reported for the first time in our case.

## INTRODUCTION

Pectus excavatum (PE) is the depression of the lower part of manubrium sterni and xiphoid process. It is the most common anterior chest wall deformity. It’s incidence is 1 in 400 to 1.000 live births and it is three to five times more common in males^(1)^. PE is usually sporadic but can also be associated with connective tissue disorders, such as Marfan syndrome and Ehlers-Danlos syndrome, neuromuscular disease, and a variety of other genetic conditions, including Noonan syndrome and Turner syndrome.

The main problem of PE depends on the cardiopulmonary morbidity caused by the narrowing of the thoracic space. The severity of the chest wall deformity determines the morbidity. Another problem with PE is the psychosocial problems caused by the cosmetic concerns.

One third of PE patients present during infancy^(2)^. Spontaneous regression is rare and progression may continue until the end of adolescence^(3)^. The main symptoms of these patients are exertional intolerance, chest pain, and shortness of breath. Respiratory problems may even begin during infancy.

Until now, prenatal diagnosis of this deformity has been reported only once by Salamanca et al.^(4)^ in two patients who postnatally diagnosed as having Down syndrome. In this report, we present another case which we diagnosed as PE during a second-trimester fetal anatomic scan.

## CASE REPORT

**Figure 1:**
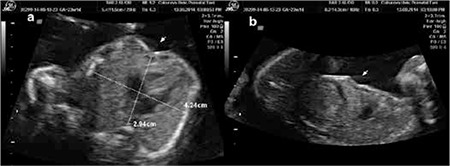
Ultrasonographic images of pectus excavatum, a) Axial thoracic section, pectus severity index is calculated by the ratio of the lateral diameter of the chest to the distance between the sternum and spine at the point of maximal depression (pectus severity index: 4.24 cm/2.94 cm=1.44), b) Sagittal thoracic section (arrows indicate the defect)

**Figure 2:**
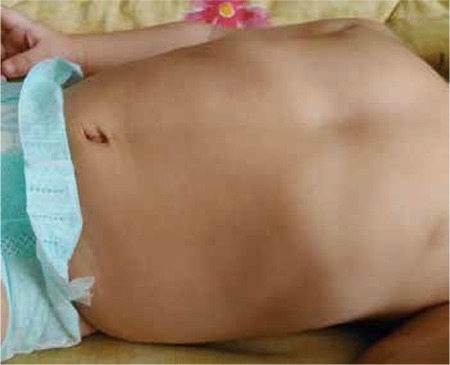
Postnatal photograph of the child with pectus excavatum

A pregnant woman aged 35 years presented during her 23rd week of gestation for a routine fetal anatomic scan. Her medical history was not significant for any clinical condition. She had a healthy boy aged 11 years delivered by cesarean section. The ultrasound examination was performed using a Voluson 730 Pro with RAB2-5L 3D abdominal transducer (2-5 MHz) probe. Fetal biometric measures were compatible with the gestational age. The fetus had a normal anatomic scan except for the depression at the lower part of the sternum. The depression at this level was seen in both the transverse and sagittal sections of the lower thorax (Figure 1). The defect did not deteriorate the cardiac functions of the fetus. Pectus severity index (PSI) as adopted from postnatal method was calculated as 1.44 (Figure 1). There was no family history for any anterior chest wall deformity. The patient delivered a 3.100 g male baby during the 39^th^ week of gestation by cesarean section with an Apgar score of 9/10. Postnatal examination confirmed the diagnosis of PE and he had no cardiopulmonary complications during the two-year follow-up period (Figure 2). The child was otherwise healthy. Therefore, surgical correction of the deformity was postponed until after the end of adolescence.

## DISCUSSION

PE is an anterior chest wall deformity that primarily compromises cardiopulmonary functions in severe cases. However, cosmetic problems are the major problem for most patients. Currently, surgery is the only treatment approach for these patients. Severe chest wall restriction could initiate symptoms during infancy and may require surgical treatment during this period. For other patients, surgery is usually postponed until the end of adolescence, because the deformity may worsen during the growth spurt of adolescence.

Severity of PE is defined by PSI, which is measured using a computerized tomography scan of the thorax^(5)^. PSI is the ratio of the lateral diameter of the chest to the distance between the sternum and spine at the point of maximal depression^(5)^. Surgery is offered to patients with a PSI of >3.25^(5)^. Prenatal adaption of this index is reported for the first time in our case. The PSI of our fetus (1.44) was consistent with good prognosis (PSI<3.25), which can be defined as deferability of surgery at least until the end of adolescence. Other tests needed for these patients are electrocardiography, echocardiography, and exercise tests in order to establish preoperative cardiopulmonary functions.

To the best of our knowledge, prenatal diagnosis of PE has been reported only once in the literature, in 1992^(4)^. In that report, two cases were described and both were later diagnosed as having Down syndrome, even though children with Down syndrome rarely have PE. In our case, PE was not associated with any genetic syndrome like Down, Turner or Noonan. Routine karyotyping may not be necessary for prenatally-diagnosed cases. Congenital cardiac anomalies (atrial/ventricular septal defects, partial atrioventricular septal defects), cardiac malpositioning or pericardial effusion are associated with PE^(6)^. Therefore, these fetuses should be examined thoroughly for any cardiac anomaly.

Consequently, chest wall examination should be incorporated into the routine fetal anatomic scan during the second trimester. Accompanying abnormal findings may require karyotyping. Patients should be counseled that this is rarely associated with genetic syndromes and prognosis depends mainly on the severity of the defect and cardiopulmonary complications it causes. PSI might be a promising marker for determining the prognosis of such fetuses if it can be supported by other studies.
